# Direct mapping of hydrangea blue-complex in sepal tissues of *Hydrangea macrophylla*

**DOI:** 10.1038/s41598-019-41968-7

**Published:** 2019-04-01

**Authors:** Takaaki Ito, Dan Aoki, Kazuhiko Fukushima, Kumi Yoshida

**Affiliations:** 1 Graduate School of Information Sciences, Nagoya University, Chikusa, Nagoya 464-8601 Japan; 2 Graduate School of Bioagricultural Sciences, Nagoya University, Chikusa, Nagoya 464-8601 Japan; 3 Graduate School of Informatics, Nagoya University, Chikusa, Nagoya 464-8601 Japan

## Abstract

The original sepal color of *Hydrangea macrophylla* is blue, although it is well known that sepal color easily changes from blue through purple to red. All the colors are due to a unique anthocyanin, 3-*O*-glucosyldelphinidin, and both aluminum ion (Al^3+^) and copigments, 5-*O*-caffeoyl and/or 5-*O*-*p*-coumaroylquinic acid are essential for blue coloration. A mixture of 3-*O*-glucosyldelphinidin, 5-*O*-acylquinic acid, and Al^3+^ in a buffer solution at pH 4 produces a stable blue solution with visible absorption and circular dichroism spectra identical to those of the sepals, then, we named this blue pigment as ‘hydrangea blue-complex’. The hydrangea blue-complex consists of 3-*O*-glucosyldelphinidin, Al^3+^, and 5-*O*-acylquinic acid in a ratio 1:1:1 as determined by the electrospray ionization time-of-flight mass spectrometry and nuclear magnetic resonance spectra. To map the distribution of hydrangea blue-complex in sepal tissues, we carried out cryo-time-of-flight secondary ion mass spectrometry analysis. The spectrum of the reproduced hydrangea blue-complex with negative mode-detection gave a molecular ion at *m/z* = 841, which was consistent with the results of ESI-TOF MS. The same molecular ion peak at *m/z* = 841 was detected in freeze-fixed blue sepal-tissue. In sepal tissues, the blue cells were located in the second layer and the mass spectrometry imaging of the ion attributable to hydrangea blue-complex overlapped with the same area of the blue cells. In colorless epidermal cells, atomic ion of Al^3+^ was hardly detected and potassium adduct ion of 5-*O*-caffeoyl and/or 3-*O*-acylquinic acid were found. This is the first report about the distribution of aluminum, potassium, hydrangea blue-complex, and copigment in sepal tissues and the first evidence that aluminum and hydrangea blue-complex exist in blue sepal cells and are involved in blue coloration.

## Introduction

**Figure 1:**
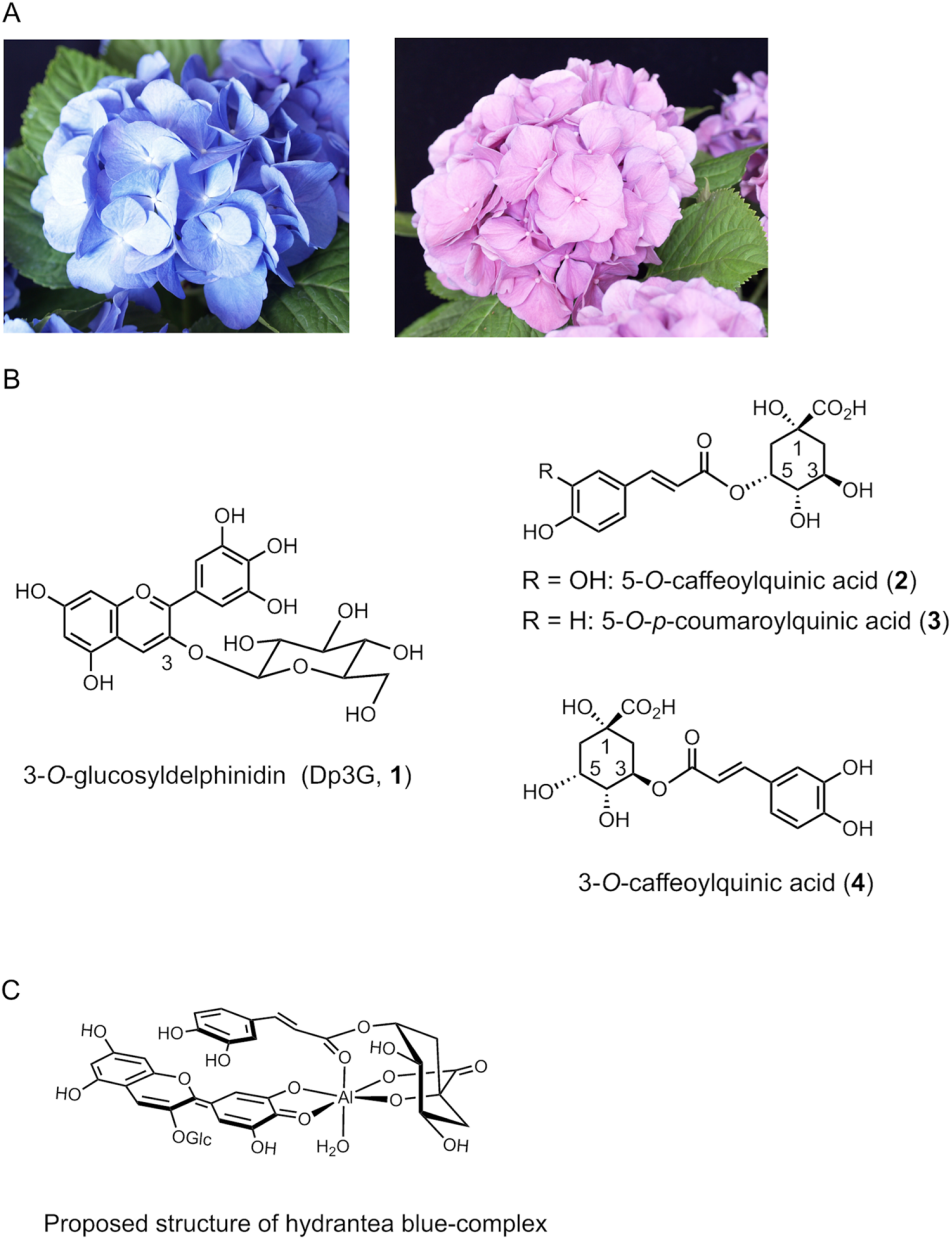
Hydrangea flower and the components responsible for sepal coloration. (**A**) Photo of hydrangea, *Hydrangea macrophylla* cv. Narumi blue (the left) and cv. Narumi red (the right). (**B**) Organic components in hydrangea sepals. (**C**) Proposed structure of hydrangea blue-complex.

The plant species *Hydrangea macrophylla* originates from Japan and East Asia. The original color of its sepals is blue (Fig. 1A, the left), but thanks to extensive breeding efforts, hydrangeas with various colors, from red (Fig. 1A, the right), to white and green are available nowadays. It is also well known that the color of *H. macrophylla* sepals change easily from red through purple to blue, depending on cultivation conditions. This phenomenon has been attracting the attention of not only plant scientists and horticulturist, but also flower lovers. The first reports about the strong correlation between soil acidity and blue color development^1^ and the role of aluminum ion (Al^3+^)^2,3^ date back to the early 19th century. In acidic soils (pH less than 5.0), the level of water-soluble Al^3+^ in soil is increased and absorbed by the roots. Once absorbed, the Al ions are transported into sepals where they form complexes with anthocyanin, resulting in blue sepal color. The structure of anthocyanins and the copigments in hydrangea sepals were reported in the mid-20th century^4–7^. Interestingly, all sepal colors originate from a single anthocyanin, 3-*O*-glucosyldelphinidin (**1**)^6^, and the same three copigment components, 5-*O*-caffeoylquinic acid (**2**), 5-*O*-*p*-coumaroylquinic acid (**3**), and 3-*O*-caffeoylquinic acid (**4**) (Fig. 1B). In 1990, the involvement of 5-*O-*acylquinic acids (**2** and **3**) in the blue sepal color development was reported, and a stable blue solution was obtained by mixing ten equivalent of Al^3+^ (1.0 mM) to **1** (0.1 mM) and **2** and/or **3** (0.1 mM)^8^. However, the molecular structure of the blue pigment and the mechanism of color variation is yet to be clarified.

Our group has studied the mechanisms of blue flower coloration and focused on the color variation of hydrangea. In 2003, we prepared blue- and red-colored protoplasts from respectively colored sepals and measured their vacuolar pH (pHv)^9^. By combining the micro-spectrophotometry and a proton-selective microelectrode analysis, we identified a significant difference in pHv between the blue and red protoplasts (4.1 and 3.3, respectively)^9^. We also quantified the levels of **1–4** and Al^3+^ in blue and red cells. The concentrations of 5-*O*-acylquinic acids (**2** and **3**) and Al^3+^ in the blue cells was significantly higher (**2** + **3**: 12.8 eq. to **1**, Al^3+^: 1 eq. to **1**) than those in the red cells (3.6 eq. and 0.03 eq., respectively)^10^. To obtain structural information of the blue complex, we synthesized various natural and unnatural copigments and attempted whether the copigments could reproduce the blue color of the sepals by mixing **1** and Al^3+^ in buffered solutions^11–13^. The essential structural elements of the copigment for blue coloration are 1-COOH, 1-OH, and 5-*O*-aromatic acyl group in the quinate^11,12^. Recently, a combination of electrospray ionization time-of-flight mass spectrometry (ESI-TOF-MS)^14^ and nuclear magnetic resonance^15^ revealed that the molecular structure of the blue metal-complex pigment in hydrangea is composed of **1** and **2** and/or **3** with Al^3+^ in the ratio 1:1:1; we named this complex “hydrangea blue-complex” (Fig. 1C)^14,15^. However, that hydrangea blue-complex exists in the blue sepal tissue is yet to be confirmed.

Imaging mass spectrometry is a powerful tool in mapping of inorganic ions and organic molecules in tissues. The most promising technique for *in planta* visualization of such water-soluble chemicals contained in vacuoles is the cryo-time-of-flight secondary ion mass spectrometry (cryo-TOF-SIMS) of freeze-fixed samples^16–20^. We have previously analyzed the distribution of Al and other inorganic ions in the stem of *H. macrophylla* and clarified the change in metal ions by treatment with aluminum salt^21^. In the present study, we carried out a molecular and atomic ion mapping of freeze-fixed sepal tissue of blue hydrangea and reported on the distribution of hydrangea blue-complex, aluminum ion, and copigments in blue cells of the sepals.

## Results and Discussion

### Concentration of organic and inorganic components in blue sepal tissue

Initially, we analyzed the organic and inorganic components involved in the blue coloration of hydrangea sepals to confirm their chemical composition (Table 1). The blue and red sepals were extracted and the content of 3-*O*-glucosyldelphinidin (**1**), 5-*O*-caffeoylquinic acid (**2**), 5-*O*-*p*-coumaroylquinic acid (**3**), and 3-*O*-caffeoylquinic acid (**4**) were quantified by using high performance liquid chromatography (HPLC). Aluminum (Al) and other inorganic atoms (sodium [Na], magnesium [Mg], pottasium [K], calcium [Ca], and iron [Fe]) were quantified by inductively coupled plasma atomic emission spectroscopy (ICP-AES) using wet-ashed sepal tissues.

**Table 1:** Content of organic and inorganic components in sepal tissues of blue and red hydrangea.

	Contents [mg/g FW] mean ± 2SE	
	Blue sepal^a^	Red sepal^b^
Dp3G (**1**)	0.48 ± 0.09	0.50 ± 0.08
5CQ (**2**)	2.3 ± 0.30	2.4 ± 0.33
5*p*CQ (**3**)	1.1 ± 0.30^*^	0.55 ± 0.07^*^
3CQ (**4**)	5.5 ± 0.82	4.5 ± 0.25
Na	n.d.	n.d.
Mg	0.19 ± 0.032	0.23 ± 0.027
Al	0.12 ± 0.029^***^	0.0061 ± 0.0029^***^
K	1.1 ± 0.14	1.1 ± 0.098
Ca	0.52 ± 0.077^**^	0.77 ± 0.11^**^
Fe	0.0011 ± 0.00061	0.0024 ± 0.0018

^a^*n* = 5 for analysis of **1–4** and *n* = 8 for analysis of inorganic elements.

^b^*n* = 3 for analysis of **1–4** and *n* = 6 for analysis of inorganic elements.

Significant differences were observed between the same marks as *p < 0.05, **p < 0.01, and

***p < 0.001.

Dp3G: 3-*O*-glucosyldelphinidin, 5CQ: 5-*O*-caffeoylquinic acid, 5*p*CQ: 5-*O*-*p*-coumaroylquinic acid, 3CQ: 3-*O*-Caffeoylquinic acid.

The contents of organic components (**1–4**) were shown in Table 1. The content of **1** in both blue and red sepals was relatively lower than our previous quantification results in colored cells^10^. This was because that anthocyanin exists only in the colored cells located at the second layer of the sepal tissue, but the quantified value in the experiments is the content in the whole sepal tissue including colorless cells^9,10^. In the previous study, we analyzed collected colored cells prepared from blue and red colored sepals. However, in this study we used the whole sepal tissue of the same species of cv. Narumi blue and Narumi red, because the purpose of the study is mapping the organic and inorganic components in tissue. These differences may be the reasons that no significant differences were observed between the quantitative data of organic components.

The results of the elemental analysis of inorganic atoms by ICP-AES are shown in Table 1. Al is known to be a toxic element for plants but, the level of Al in blue sepals was 120 ± 29 μg g^−1^ fresh weight [FW] being significantly higher than that in red sepals (6.1 ± 2.9 μg g^−1^ FW). The other elementals, such as K, Mg, and Fe, were present at usual levels and they did not differ between the blue and red tissues. The exception was Ca, whose concentration differed significantly between the two types of sepals; its content in red tissues was 770 ± 110 μg g^−1^ FW and in blue tissues it was 520 ± 77 μg g^−1^ FW. These results were consistent with our previous results reported for the hydrangea stem^21^.

### Reproduction of the blue color and mass spectrometry analysis of the solutions

**Figure 2:**
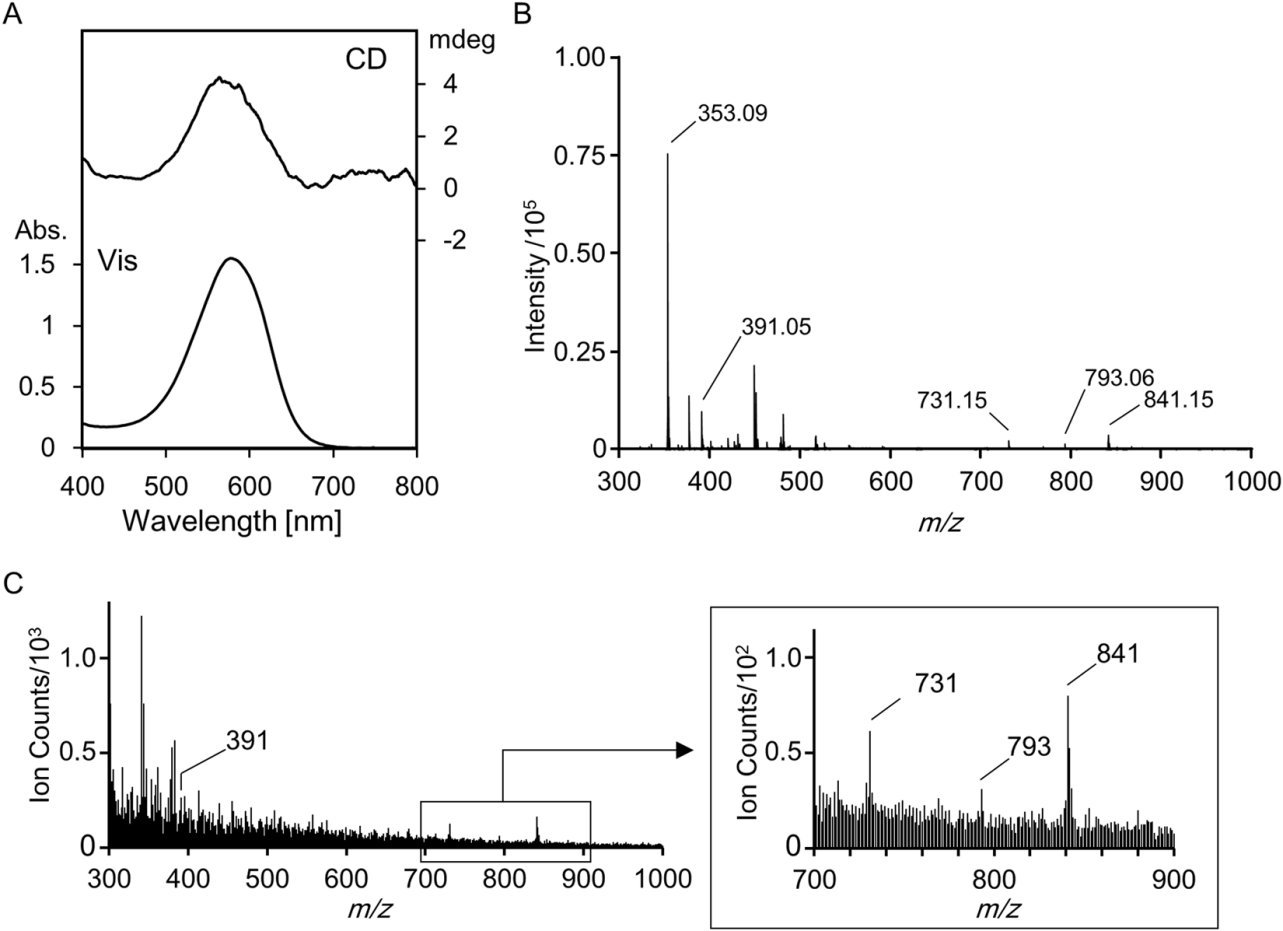
Spectroscopic analysis of the reproduced hydrangea blue-complex. (**A**) Visible and CD spectra of reproduced hydrangea blue-complex mixed with **1** (100 μM), **2** (200 μM), and AlCl_3_ (100 μM) in 100 μM KCl-H_2_O. (**B**) ESI-TOF MS spectrum of the hydrangea blue-complex conducted in negative detection mode. (**C**) Cryo-TOF-SIMS spectrum of hydrangea blue-complex conducted in negative detection mode.

Before conducting the cryo-TOF-SIMS of the sepal tissue, we analyzed the reproduced hydrangea blue-complex and other standard solutions by cryo-TOF-SIMS to test whether this method can be applied to analyze the complex and give the same molecular ions as detected with ESI-TOF MS analysis^14^. 3-*O*-Glucosyldelphinidin (**1**, 100 μM), 5-*O*-caffeoylquinic acid (**2**, 200 μM), and AlCl_3_ (100 μM) were dissolved in 100 μM KCl aqueous solution and measured using visible adsorption (Vis) spectrum and circular dichroism (CD) (Fig. 2A). The pH of the solution was approximately 3.7 and its color was blue. The Vis and CD spectra were the same as those reported previously^11–14^, indicating that hydrangea blue-complex was reproduced accurately.

The solution was analyzed by ESI-TOF MS using negative (Fig. 2B) and positive detection modes (Fig. S1A). The molecular ion peaks at *m/z* = 841 and *m/z* = 843 were detected with the negative and positive mode, respectively, confirming that the complex composed of **1**, **2**, and Al^3+^ in the ratio of 1:1:1 existed in the solution^14^.

Next, the solution of hydrangea blue-complex was poured into the sample vessel, then frozen, and analyzed by the cryo-TOF-SIMS. As shown in Fig. 2C, the negative detection mode identified a molecular ion peak at *m/z* = 841 ([**1** + Al + **2**−4 H]^−^), which was attributable to the molecular ion of hydrangea blue-complex. This result confirmed that SIMS measurements could identify the same molecular ion of the hydrangea blue-complex as that detected by the ESI-TOF-MS analysis. However, such ion could not be detected with the positive detection mode (Fig. S1B). Therefore, we selected the ion peak at *m/z* = 841 in negative detection mode to visualize the hydrangea blue-complex distribution.

In addition to the molecular ion peak of hydrangea blue-complex, the negative detection spectrum gave molecular ion peaks at *m/z* = 793 and *m/z* = 731, which are attributable to [2 × **2** + 2 × Al + K−8 H]^−^ and [2 × **2** + Al−4 H]^−^, respectively (Fig. 2C). Potassium adduct of **2** was also observed at *m/z* = 391 [**2** + K−2 H]^−^ (Fig. 2C), although the intensity of the ion is not so high. This might because co-existing with aluminum ion decreased the ion at *m/z* = 391 and increased the ions at *m/z* = 793 and *m/z* = 731 (Fig. S2C,D). The positive detection mode of the hydrangea blue-complex solution produced only a peak at *m/z* = 393 [**2** + K]^+^ (Fig. S1B).

The same ions derived from the copigment were detected when a solution obtained by mixing **2** (200 μM) and AlCl_3_ (100 μM) in 100 μM aqueous KCl was measured using cryo-TOF-SIMS with negative detection mode (Fig. S2A). Aluminum and potassium were measured with positive mode as monovalent ions [Al]^+^ and [K]^+^, respectively.

### Cryo-TOF-SIMS imaging of the blue sepal tissue

**Figure 3:**
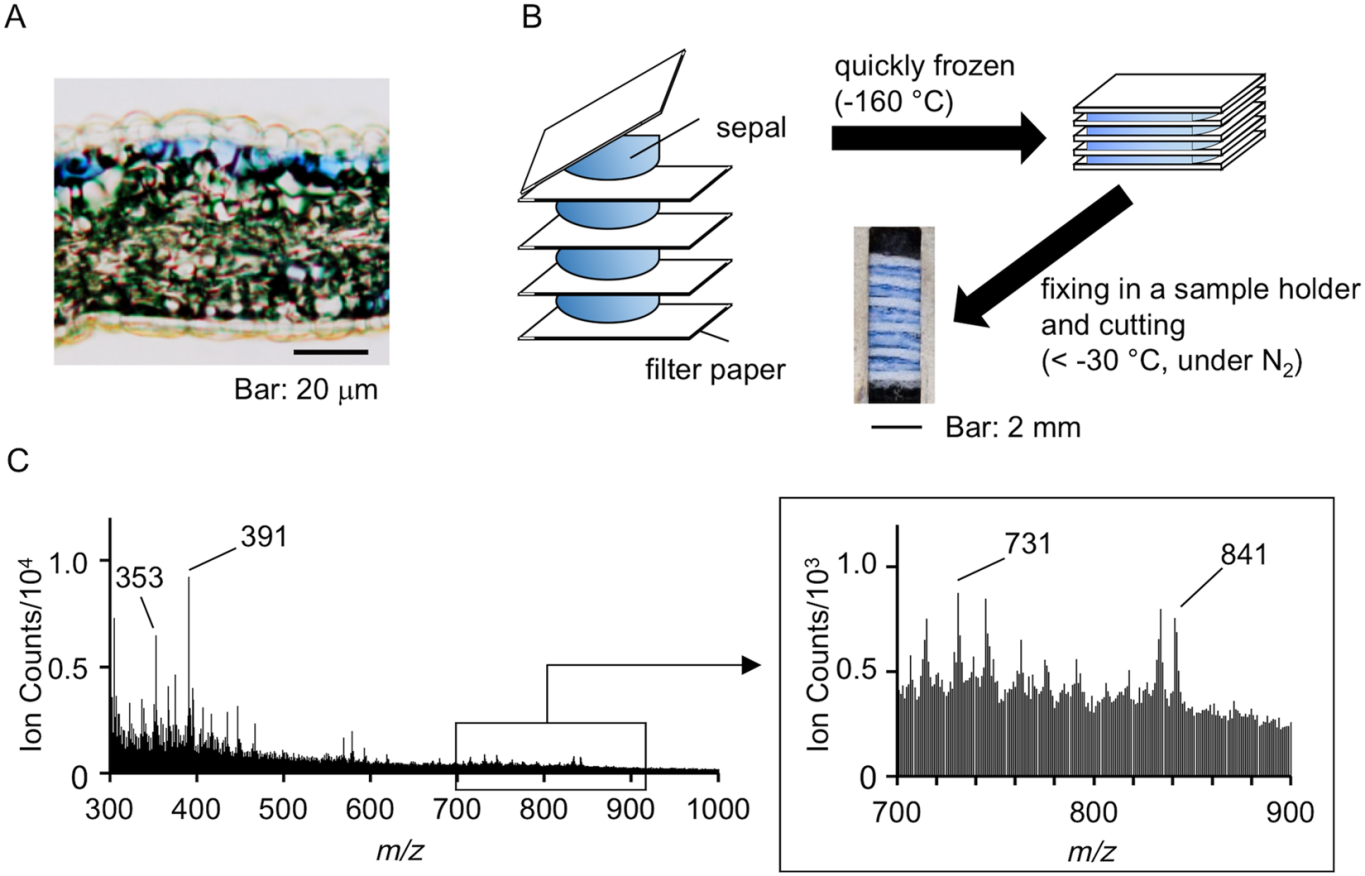
Cryo-TOF-SIMS analysis of the blue sepal tissue. (**A**) Microscopic observation of the transverse section of the blue sepal. (**B**) Preparation of blue sepal sample for cryo-TOF-SIMS. (**C**) Negative cryo-TOF-SIMS spectrum of the transverse surface of the blue sepal tissue.

After detecting the molecular ion by negative cryo-TOF-SIMS spectra of the reproduced hydrangea blue-complex solution, we examined the presence of the molecule in blue sepal tissues. The transverse section of the blue sepal tissue was observed under a microscope (Fig. 3A). In hydrangea sepals, colored cells are located in the second layer of the epidermis and epidermal cells are colorless^9^. This distribution of colored cells differs from that found in typical flower tissues^22^.

The blue hydrangea sepals were cut into approximately 1 cm square pieces and placed between filter papers that were stacked on each other. The sandwich-structured sample was quickly frozen with Freon^®^ 22 at −160 °C (Fig. 3B). In the sample holder, the adaxial epidermis of the sepals was facing upward, and the abaxial epidermis downward. After the surface of the tissues was cut to obtain a fresh transverse surface, the sample in the holder was transferred to the SIMS stage to collect the negative and positive cryo-TOF-SIMS spectra at −120 °C. The total ion spectrum in the negative mode is shown in Fig. 3C. In the negative spectrum, the molecular ions were detected at *m/z* = 841: [**1** + Al + **2**−4 H]^−^; 731: [2 × **2** + Al−4 H]^−^; 391: [**2** + K−2 H]^−^; and 353 [**2**−H]^−^. This is the first report of the detection of molecular ion peaks of hydrangea blue-complex and other copigments in blue sepal tissue by cryo-TOF-SIMS. The two copigments **2** and **4** are stereoisomers and therefore have the same molecular weight; the ions at *m/z* = 391 and 353 could not be distinguished from each other. However, we recently reported that **2** gave a complex with Al ion, whereas **4** did not^23^. Therefore, the ion at *m/z* = 731 should be composed of **2** and Al ion. In contrast, in the positive spectra, no molecular ions of hydrangea blue-complex and other complexes with copigments were observed, and only a potassium adduct of **2** ([**2** + K]^+^) was detected at *m/z* = 393 (Fig. S3A). The same measurements were carried out using red sepals. No molecular ion was detected at *m/z* = 841, and only ions at *m/z* = 391: [**2** + K−2 H]^−^ and 353 [**2**−H]^–^ were detected in the negative mode (Fig. S3B). The positive mode detection produced no assignable molecular ions (Fig. S3C).

**Figure 4:**
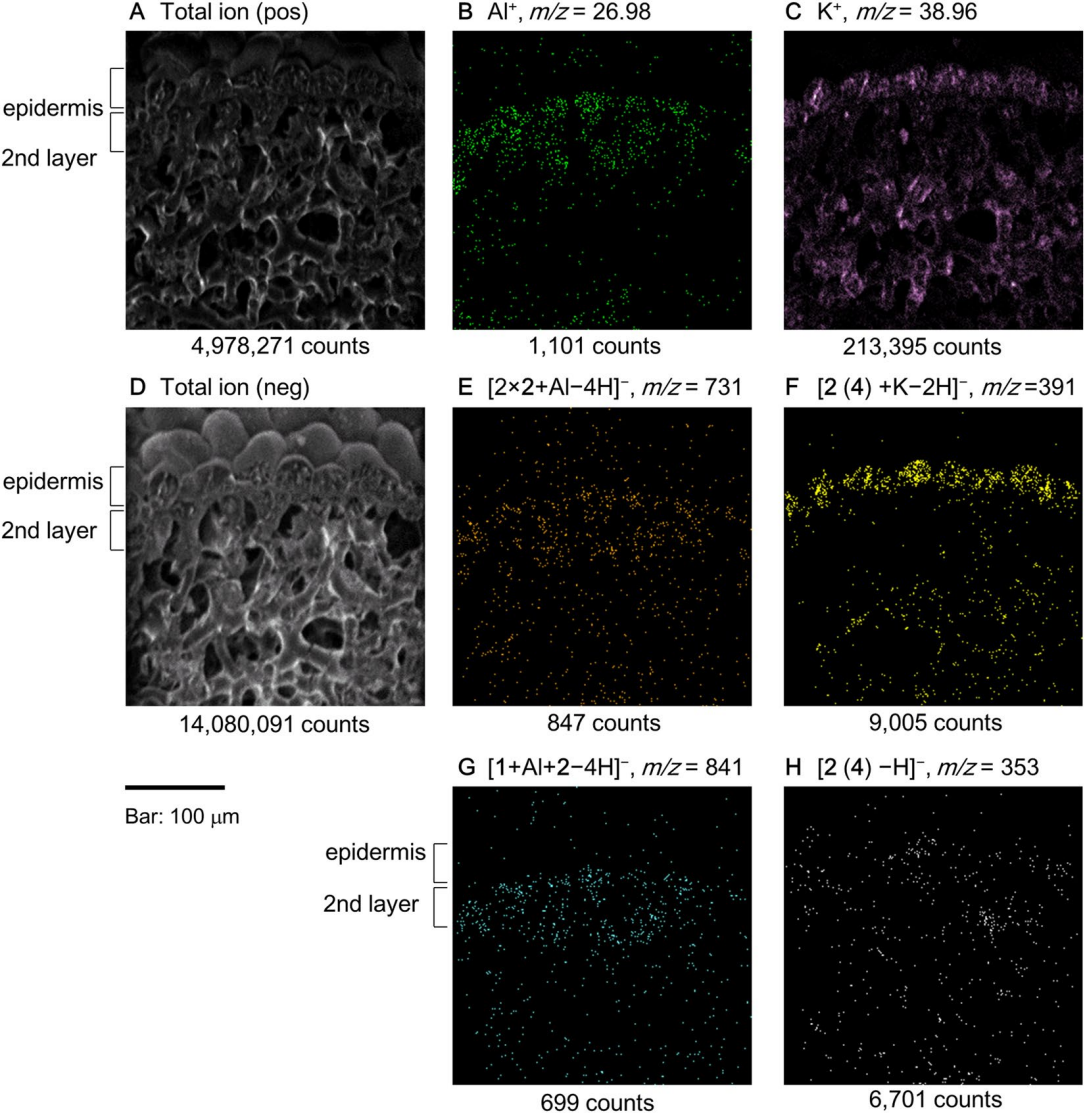
Cryo-TOF-SIMS ion images of the transverse surface of blue sepal tissue. (**A**) Total ion distribution inferred by positive mode detection. (**B**) Al^+^. (**C**) K^+^. (**D**) Total ion distribution obtained by negative detection mode. (**E**) *m/z* = 731: [2 × **2** + Al–4 H]^−^. (**F**) potassium adduct of 5-*O*-caffeoylquinic acid and/or 3-*O*-caffeoylquinic acid (*m/z* = 391: [**2** (**4**) + K−2 H]^−^). (**G**) hydrangea blue-complex (*m/z* = 841: [**1** + Al + **2**−4 H]^−^). (**H**) 5-*O*-caffeoylquinic acid and/or 3-*O*-caffeoylquinic acid (*m/z* = 353: [**2** (**4**) −H]^–^).

Next, we mapped the detected ions of the blue sepal tissue (Fig. 4). Figure 4A shows the total ion image of positive detection and Fig. 4D is that of negative detection. Those images corroborate the microscopic observations of the transverse sections of the hydrangea sepal tissues. The map of [Al]^+^ and [K]^+^ is shown in Fig. 4B,C, respectively, indicating that Al is distributed in the second layer and K is found throughout the sepal tissue. The molecular ion at *m/z* = 731, which is attributable to [2 × **2** + Al−4 H]^−^, showed a similar distribution to that of [Al]^+^ (Fig. 4E), whereas the distribution of the ion at *m/z* = 391, which is attributed to [**2** + K−2 H]^−^, differed as the ion was localized mainly in the surface epidermal cells (Fig. 4F). The distribution of the ion at *m/z* = 841 (Fig. 4G), which was identified as hydrangea blue-complex, overlapped with that of [Al]^+^ and the Al complex of 2 ([**2** × **2** + Al−4 H]^−^). The molecular ion at *m/z* = 353 identified as **2** or **4** was distributed in both the epidermis and the second cell layer (Fig. 4H).

The mapping results indicated that each organic and inorganic component had a different distribution in the sepal tissue. Aluminum was not distributed in the epidermal cells; it was mostly contained in the second cell layer (Fig. 4B). This is the first time that toxic Al was not observed in the surface cells, although the mechanism and reason for such a distribution remains to be clarified. The distribution of Al complexes, hydrangea blue-complex (Fig. 4G) and the copigment-Al complex (Fig. 4E), coincided with that of Al. This distribution was expected and consistent with the localization of the blue-colored cells in blue sepals (Fig. 3A). In contrast, the distribution of K ion was not confined to a specific tissue, but it was found in all the tissues (Fig. 4C). This might be due to its essential role in the control of osmotic pressure in plant cells. The copigments **2** and/or **4** were distributed throughout the tissue in different forms: in the epidermal cells they existed as potassium salts, because no Al ions existed. In contrast, in the inner cells both K and Al ions exist, but affinity of Al ions to oxygen atom is stronger than that of K ions, therefore, copigments may be detected as Al complexes.

**Figure 5:**
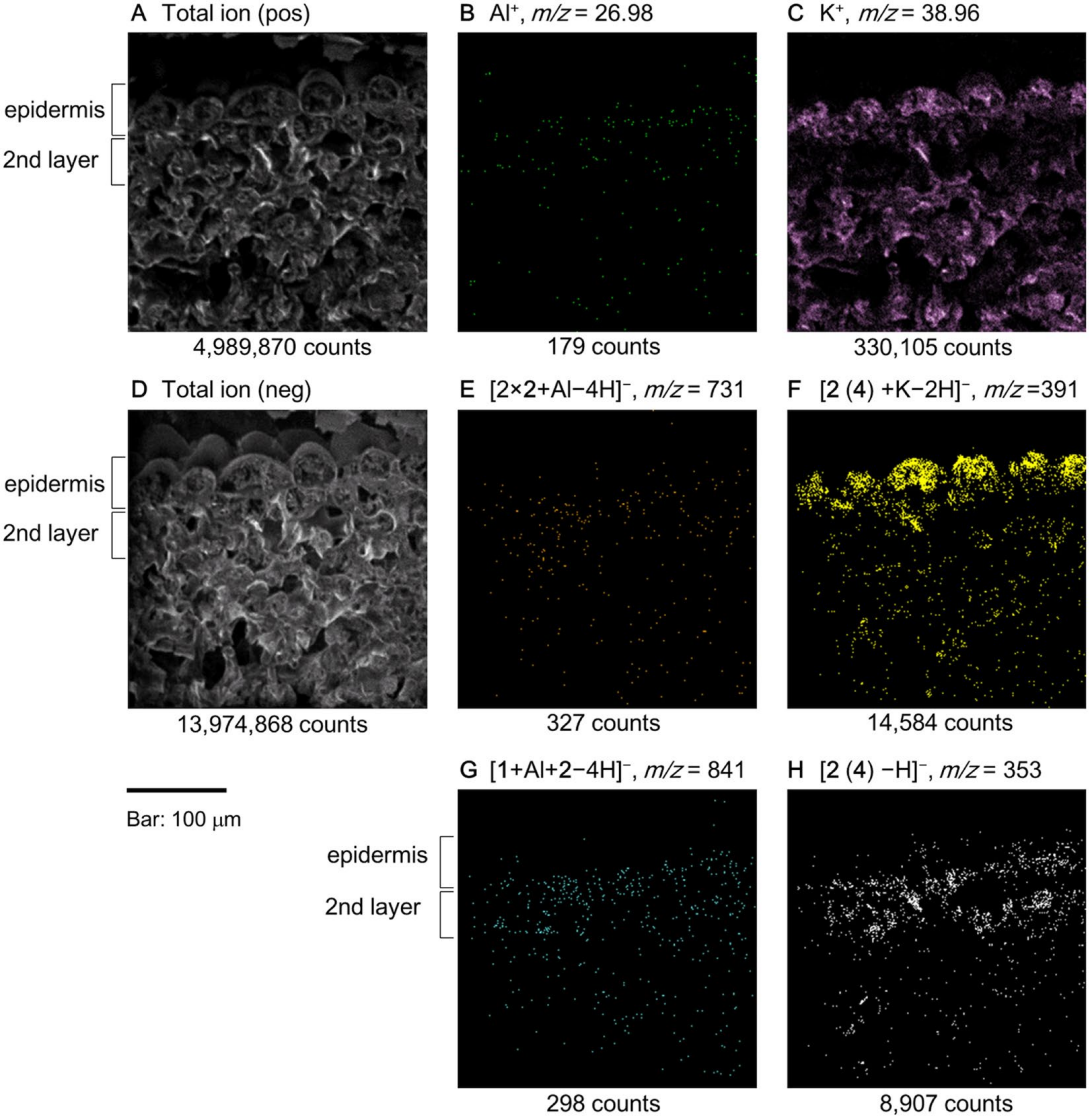
Cryo-TOF-SIMS ion images of the transverse surface of red sepal tissue. (**A**) Total ion distribution by positive mode detection. (**B**) Al^+^. (**C**) K^+^. (**D**) Total ion distribution by negative mode detection. (**E**) *m/z* = 731: [2 × **2** + Al–4 H]^−^. (**F**) Potassium adduct of 5-*O*-caffeoylquinic acid and/or 3-*O*-caffeoylquinic acid (*m/z* = 391: [**2** (**4**) + K−2 H]^−^). (**G**) Hydrangea blue-complex (*m/z* = 841: [**1** + Al + **2**−4 H]^−^). (**H**) 5-*O*-Caffeoylquinic acid and/or 3-*O*-caffeoylquinic acid (*m/z* = 353: [**2** (**4**) −H]^–^).

The mass imaging of the red sepal tissue is shown in Fig. 5. The results were significantly different from those reported for the blue sepal tissue. As shown in Fig. 5B, [Al]^+^ was nearly absent, which was consistent with the results of the elemental analysis by ICP-AES. Similar to the mapping data of [Al]^+^, the molecular ions at *m/z* = 841 and *m/z* = 731 were hardly detected in red tissues (Fig. 5G and E, respectively). However, the distribution of [K]^+^ was similar to that in blue sepal tissue (Fig. 5C), and the localization of K adduct of **2** or **4** (*m/z* = 391) was observed clearly in the epidermal cells (Fig. 5F) in the same pattern as that of the blue sepal tissue (Fig. 4F). In regard to the ion attributable to a copigment (*m/z* = 353), a higher ion count was detected in red sepal tissue than in blue sepals (Fig. 5H).

The ionization efficiency of each molecular and metal ion differs and is affected by the coexisting components and ion strength of the sample in question. Therefore, the mass imaging data are not suitable for accurate quantitative analysis of ions. However, when combined with chemical quantification (Table 1), it produced a clear and accurate differentiation of the localization of Al ion and Al complexes between blue and red tissues. The content of Al in blue sepals was 0.12 mg/g FW but in red sepals the content was approximately 1/20 (Table 1). This large difference of Al content between blue and red sepals was reflected in the numbers of ion counts in mass-imaging results of Fig. 4B (1,101) and Fig. 5B (179). In contrast, the content of K ion was almost the same in blue and red sepals, then, the ion counts of [K]^+^ in mass-imaging results (Figs. 4C, 5C) indicates no typical difference as 213,395 in blue tissue and 330,105 in red tissue. The contents of **1**, **2** and **4** did not differ between blue and red sepals (Table 1), but the difference in Al ion contents between blue and red tissues brought difference in ion counts of *m/z* = 731 (Figs. 4E, 5E) and 841 (Figs. 4G, 5G).

In the fully colored hydrangea sepal tissues, more than 95% of the cell volume is occupied with vacuoles, the organelles where secondary metabolites and inorganic ions are located^9,10,22^. Thus, the difference in distribution of inorganic elements and organic molecules observed in this study indicates that the distribution of each component in the sepal vacuoles is specific to the component. In conclusion, we have confirmed that hydrangea blue-complex is distributed in the blue cells of the sepals and it is involved in blue coloration. Using the cryo-TOF-SIMS analysis, we provide the first evidence of the clear differences in Al ion distribution in sepal tissues. Further studies, which are underway, will examine the mechanisms of localization of Al and other organic components.

## Methods

### Plant material

*H. macrophylla* cv. Narumi blue and cv. Narumi red were donated by the Okumura-Seika-en (Toyoake, Japan) and cultivated in the Botanical Garden, Nagoya University Museum. Fresh, fully colored sepals were collected and cut into approximately 10 mm square pieces. The tissues were quickly frozen in liquid Freon® 22 (DuPont, Wilmington, DE, USA) at −160 °C and stored at −80 °C until use.

### Chemical reagents

Chemicals used in this study were the same as previously reported^14^. Briefly, 3-*O*-glucosyldelphinidin (Dp3G; **1**) was purified from the seed coat of the scarlet bean, *Phaseolus coccineus*, and the copigments 5-*O*-caffeoylquinic acid (**2**) and 5-*O*-*p*-coumaroylquinic acid (**3**) were synthesized^13^. 3-*O*-Caffeoylquinic acid (**4**) and trifluoroacetic acid (TFA) were purchased form FUJIFILM Wako Pure Chemical Corporation (Osaka, Japan), and KCl and AlCl_3_**·**12H_2_O was purchased from KANTO KAGAKU (Tokyo, Japan).

### Reproduction of the hydrangea blue-complex

The reproduction of the blue solution contained hydrangea blue-complex was conducted as reported previously with some modifications^14^. Briefly, stock solutions of Dp3G (**1**, 1 mM), copigment (**2**, 2 mM), AlCl_3_ (1 mM), and KCl (1 mM) were mixed in a 1.5 mL microtube at a final concentration of 100 μM (**1**, AlCl_3_ and KCl) and 200 μM (**2**). The pH of the solution was measured with a D-21 pH meter and a LAQUA 9618S-10D electrode (HORIBA Instruments, Kyoto, Japan).

### Measurements of Vis and CD spectra

Vis spectra were recorded with a UV V-550 spectrophotometer (JASCO International Co., Ltd., Tokyo, Japan) from 400 to 800 nm at a scanning rate of 400 nm min^−1^ and 25 °C. CD spectra were measured with a CD J-720 spectrophotometer (JASCO) over the range of 400–800 nm with a scanning rate of 500 nm min^−1^ at 25 °C; the mean of four trials was determined. The reproduced blue solution was measured in a quartz cell with a 10 mm path length.

### HPLC analysis of organic components in sepals

Quantification of organic components by HPLC was carried out as previously described^24^ with a slight modification. Briefly, about 250 mg of tissue samples were extracted with 50% aqueous acetonitrile (CH_3_CN) solution containing 5.0% TFA for four times (1.5 mL × 4) and the extracts were combined and messed up to 10 mL. The extract was analyzed with HPLC system composed of two PU-1580 pumps, a HG-1580-32 mixer, a DG-1580-53 degasser, an MD-2018 detector, and CO-1565 column oven, and the system was controlled using ChromNAV ver 2 software. A reversed phase column (Develosil ODS-HG-5, 2.0 mm i.d. × 250 mm, Nomura Chemicals, Seto, Japan) were eluted with a linear gradient elution from 10% to 18.8% aqueous CH_3_CN solution containing 0.5% trifluoroacetic acid for 30 min with a flow rate of 0.2 mL min^−1^ at 40 °C. The calibration curves of each component were obtained with triplicate analyses of the standard solutions.

### ESI-TOF-MS analysis

ESI-TOF-MS analysis was performed using a micrOTOF-QII mass spectrometer (Bruker, Billerica, MA, USA) and analyzed using the included software^14^.

### ICP-AES analysis of sepals

About 20 mg of tissue samples were analyzed by ICP-AES as previously described^21^. Briefly, frozen samples were pulverized in liquid N_2_ and kept immersed in 1.5 mL 60% HNO_3_ at room temperature for 16 h. The sample suspension was subsequently subjected to wet ashing under the following conditions: 105 °C for 2 h, 160 °C for 16 h with 0.2 mL of 30% H_2_O_2_. After cooling down the solution was messed up to 10 g and then filtered. The resultant sample solutions were analyzed using an ICP instrument (Vista-Pro, Seiko Instruments/Varian Instruments, Chiba, Japan). The detection limits of each element were described in Table S1. The concentration of each inorganic metal was quantified using a calibration curve for the standard solution (ICP multi-element standard solution IV, Merck, Darmstadt, Germany). Resultant data were expressed as the mean ± 2 × standard error (2 S.E.)

### Cryo-TOF-SIMS analysis

The cryo-TOF-SIMS analysis was conducted using the manufactured system containing cryo-TOF-SIMS, cryo glove box, and cryo-vacuum transfer shuttles^25,26^. The frozen sample was fixed in a sample holder, cut in the glove box (<−30 °C) under a dry N_2_ atmosphere, and transferred to the cryo-TOF-SIMS (TRIFT III, ULVAC-PHI Inc., Kanagawa, Japan). The measurement conditions were as follows: primary ion, 22 keV Au_1_^+^ (5 nA); image size, 300 × 300 µm and 256 × 256 pixels; pulse width, 1.8 ns (bunched for spectrum) or 13.0 ns (non-bunched for image); temperature, −120 °C; measurement time, 10–15 min. Bunched and non-bunched images were obtained in positive and negative ion detection modes, respectively. Calibration curves used were water clusters as follows. Positive: [CH]^+^ 13.0078, [(H_2_O)_2_ + H]^+^ 37.0289, [(H_2_O)_4_ + H]^+^ 73.0500, [(H_2_O)_11_ + H]^+^ 199.1238, negative: [OH]^−^ 17.0027, [(H_2_O)_3_-H]^−^ 53.0238, [(H_2_O)_4_-H]^−^ 71.0343, [(H_2_O)_11_-H]^−^ 197.1081. A low-energy pulsed electron gun (30 eV) was used for surface charge compensation. TOF-SIMS data analysis was carried out using WinCadence software (ULVAC-PHI. Inc.) and the color scales of the obtained bitmap images were changed in ImageJ software^27^.

### Microscopic observations

Thin transverse sections (5 μm thick) were prepared following the previously reported method for preparing hard tissue sections^9^. The prepared sections were observed under an optical microscope (BX50; Olympus Corp., Tokyo, Japan).

## Supplementary information

Supporting information
