# Supplementary material for: Direct mapping of hydrangea blue-complex in sepal tissues of Hydrangea macrophylla
Source: Sci Rep. 2019 Apr 1;9:5450. doi: 10.1038/s41598-019-41968-7 (PMC6443790; doi:10.1038/s41598-019-41968-7)
Supplement: Supplementary file 1 — Supporting information [file 41598_2019_41968_MOESM1_ESM.pdf]

Supporting information

Direct mapping of hydrangea blue-complex in sepal tissues of *Hydrangea macrophylla*

Takaaki Ito[1], Dan Aoki[2]*, Kazuhiko Fukushima[2] and Kumi Yoshida[3*]

[1]Graduate School of Information Sciences, Nagoya University, Chikusa, Nagoya 464-8601, Japan

[2]Graduate School of Bioagricultural Sciences, Nagoya University, Chikusa, Nagoya 464-8601, Japan

[3]Graduate School of Informatics, Nagoya University, Chikusa, Nagoya 464-8601, Japan

*To whom correspondence should be addressed.

E-mail: daoki@agr.nagoya-u.ac.jp, Tel; +81-52-789-4062, Fax; +81-52-789-4163

E-mail: yoshidak@i.nagoya-u.ac.jp, Tel & Fax; +81-52-789-5638

Table S1. Detection wavelength and limits of ICP-AES.

Elements	Wavelength [nm]	Analytical detection limits [mg/g FW]
Na	589.592	0.051
Mg	279.553	0.0023
Al	396.152	0.00083
K	766.491	0.001
Ca	396.847	0.051
Fe	238.204	0.001

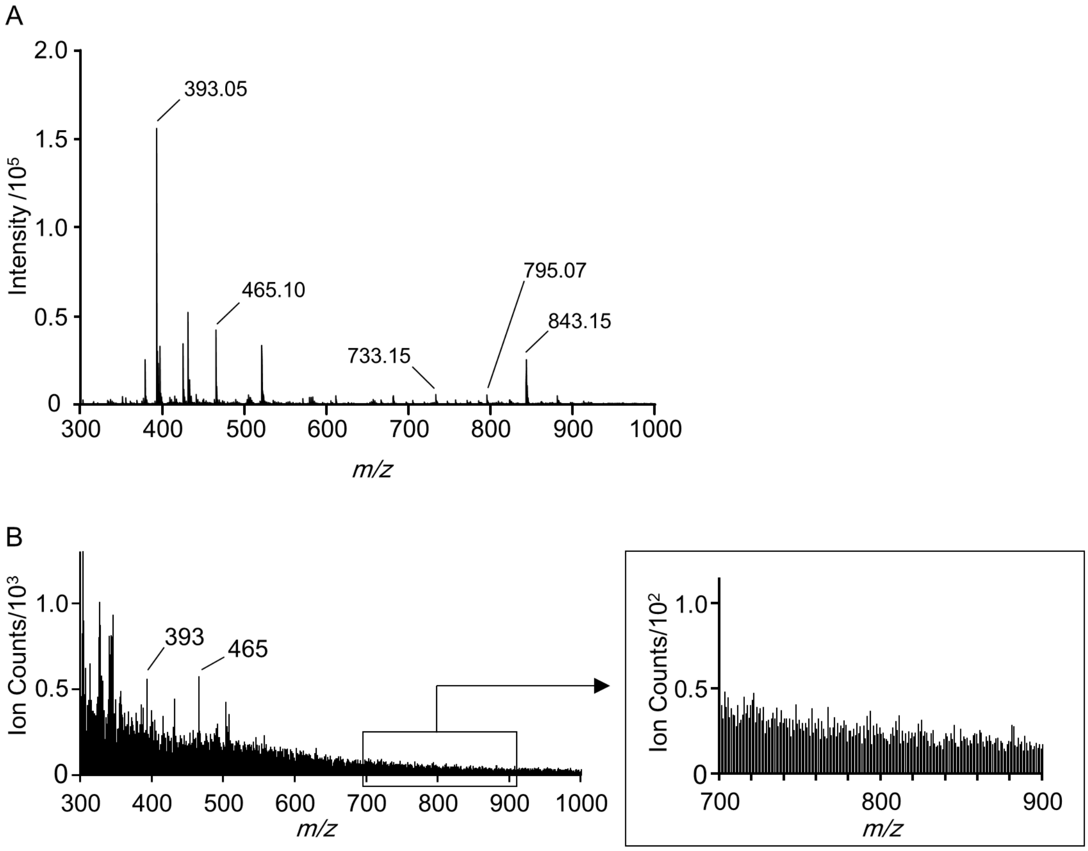

Figure S1: Positive detection mass analysis of hydrangea blue-complex obtained by mixing **1** (100 μM), **2** (200 μM), and $AlCl_3$ (100 μM) in 100 μM KCl-H_2O. A: Positive spectrum of the ESI-TOF MS of the hydrangea blue-complex. B: Positive spectrum of the cryo-TOF-SIMS of hydrangea blue-complex.

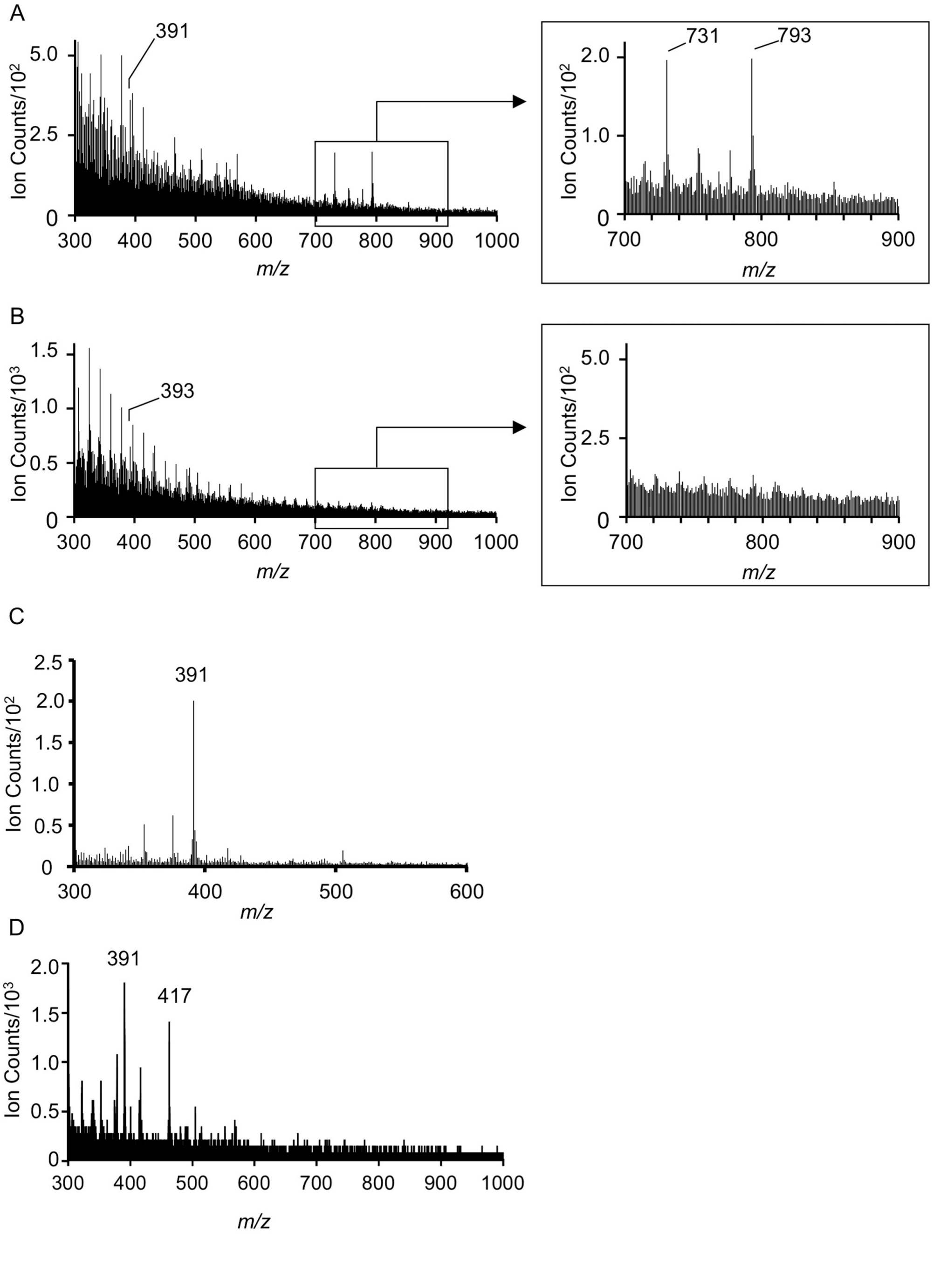

Figure S2: Cryo-TOF-SIMS spectra of standard solutions obtained by mixing **2** (200 µM), $AlCl_3$ (100 µM), and **1** (100 µM) in 100 µM KCl-H_2O. A: Cryo-TOF-SIMS spectrum of the negative detection mode of **2** (200 µM) with $AlCl_3$ (100 µM); B: Cryo-TOF-SIMS spectrum of the positive detection mode of **2** (200 µM) with $AlCl_3$ (100 µM); C: Cryo-TOF-SIMS spectrum of the negative detection mode of **2** in KCl; D: Cryo-TOF-SIMS spectrum of the negative detection mode of **2** (200 µM) and **1** (100 µM) without $AlCl_3$ in KCl.

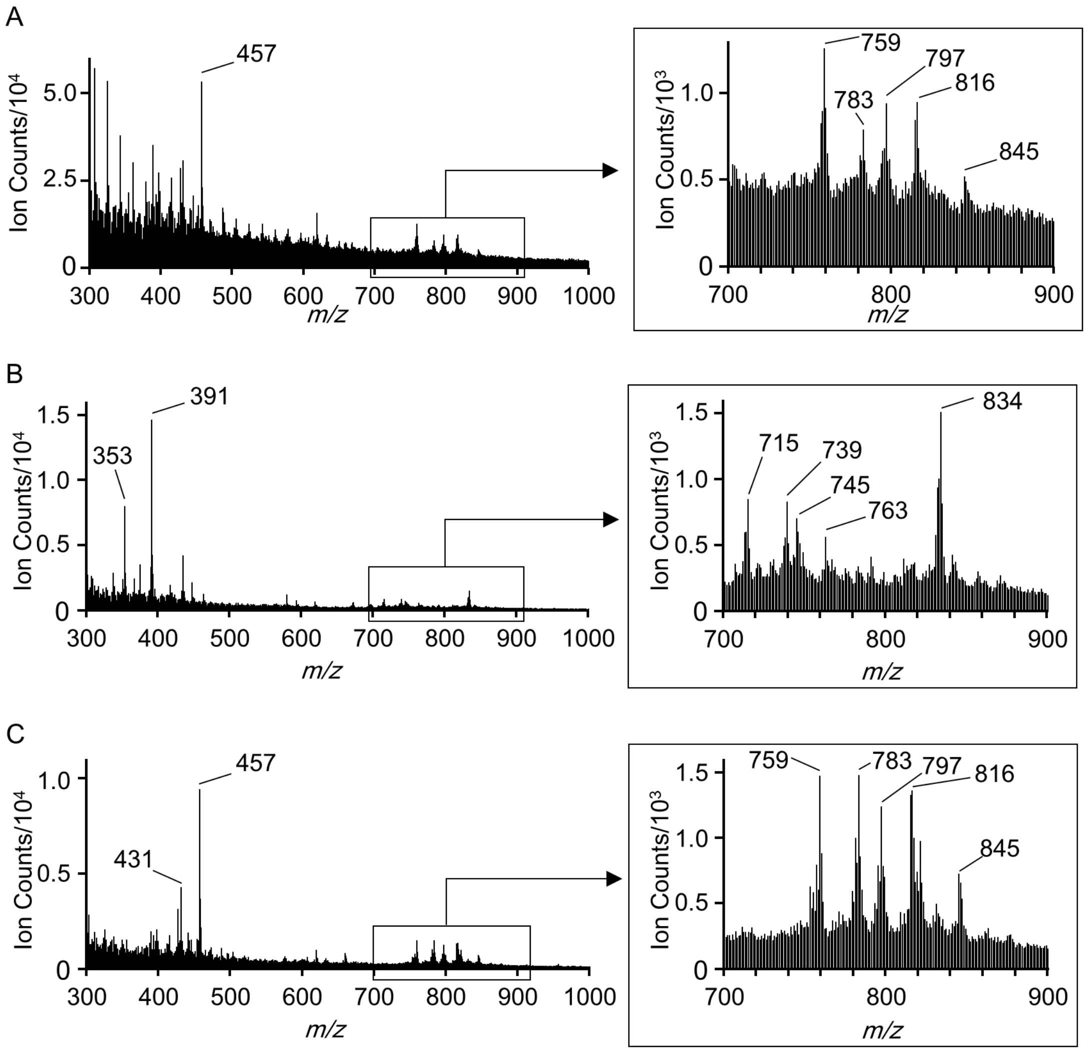

Figure S3: Cryo-TOF-SIMS spectra of blue and red sepal tissues. A: Positive cryo-TOF-SIMS spectrum of the transverse surface of blue sepal tissue. B: Negative cryo-TOF-SIMS spectrum of the transverse surface of red sepal tissue. C: Positive cryo-TOF-SIMS spectrum of the transverse surface of red sepal tissue.
